# Gelsolin-mediated activation of PI3K/Akt pathway is crucial for hepatocyte growth factor-induced cell scattering in gastric carcinoma

**DOI:** 10.18632/oncotarget.8603

**Published:** 2016-04-05

**Authors:** Baohua Huang, Shuo Deng, Ser Yue Loo, Arpita Datta, Yan Lin Yap, Benedict Yan, Chia Huey Ooi, Thuy Duong Dinh, Jingli Zhuo, Lalchhandami Tochhawng, Suma Gopinadhan, Tamilarasi Jegadeesan, Patrick Tan, Manuel Salto-Tellez, Wei Peng Yong, Richie Soong, Khay Guan Yeoh, Yaw Chong Goh, Peter E. Lobie, Henry Yang, Alan Prem Kumar, Sutherland K. Maciver, Jimmy B.Y. So, Celestial T. Yap

**Affiliations:** ^1^ Department of Physiology, Yong Loo Lin School of Medicine, NUS, Singapore; ^2^ Cancer Science Institute of Singapore, National University of Singapore (NUS), Singapore; ^3^ Genome Institute of Singapore, Agency for Science, Technology and Research (A*STAR), Singapore; ^4^ Department of Surgery, National University Health System, Singapore; ^5^ Department of Pathology and Laboratory Medicine, KK Women's and Children's Hospital, Singapore; ^6^ Duke-NUS Graduate Medical School, Singapore; ^7^ Department of Biochemistry, Yong Loo Lin School of Medicine, National University of Singapore, Singapore; ^8^ Centre for Cancer Research and Cell Biology, Queen's University Belfast, Belfast, UK; ^9^ Department of Haematology-Oncology, National University Health System, Singapore; ^10^ National University Cancer Institute, Singapore; ^11^ Department of Pathology, National University Health System, Singapore; ^12^ Department of Medicine, Yong Loo Lin School of Medicine, National University of Singapore, Singapore; ^13^ Department of General Surgery, Singapore General Hospital, Singapore; ^14^ Department of Pharmacology, Yong Loo Lin School of Medicine, National University of Singapore, Singapore; ^15^ Curtin Health Innovation Research Institute, Biosciences Research Precinct, School of Biomedical Sciences, Faculty of Health Sciences, Curtin University, Bentley WA, Australia; ^16^ Department of Biological Sciences, University of North Texas, Denton, TX, USA; ^17^ Department of Biomedical Sciences, University of Edinburgh, Edinburgh, UK

**Keywords:** gelsolin, gastric cancer, E-Cadherin, hepatocyte growth factor (HGF), cancer invasion

## Abstract

In gastric cancer (GC), the main subtypes (diffuse and intestinal types) differ in pathological characteristics, with diffuse GC exhibiting early disseminative and invasive behaviour. A distinctive feature of diffuse GC is loss of intercellular adhesion. Although widely attributed to mutations in the CDH1 gene encoding E-cadherin, a significant percentage of diffuse GC do not harbor CDH1 mutations. We found that the expression of the actin-modulating cytoskeletal protein, gelsolin, is significantly higher in diffuse-type compared to intestinal-type GCs, using immunohistochemical and microarray analysis. Furthermore, in GCs with wild-type CDH1, gelsolin expression correlated inversely with CDH1 gene expression. Downregulating gelsolin using siRNA in GC cells enhanced intercellular adhesion and E-cadherin expression, and reduced invasive capacity. Interestingly, hepatocyte growth factor (HGF) induced increased gelsolin expression, and gelsolin was essential for HGF-medicated cell scattering and E-cadherin transcriptional repression through Snail, Twist and Zeb2. The HGF-dependent effect on E-cadherin was found to be mediated by interactions between gelsolin and PI3K-Akt signaling. This study reveals for the first time a function of gelsolin in the HGF/cMet oncogenic pathway, which leads to E-cadherin repression and cell scattering in gastric cancer. Our study highlights gelsolin as an important pro-disseminative factor contributing to the aggressive phenotype of diffuse GC.

## INTRODUCTION

Gastric cancer (GC) is one of the most prevalent cancer types worldwide. Despite a trend of decreasing incidence in developed countries in recent decades [1], GC remains one of the leading causes of oncologic deaths worldwide, with approximately 11000 deaths in United States in 2014 and over 700,000 deaths per annum globally [2-4]. Similar to other solid cancers, the occurrence of metastasis is an important contributing factor for GC mortality. The generation of aggressive epithelial carcinomas with disseminative ability frequently involves the loss of tight intercellular adhesions as well as increased motility and invasiveness [5]. Histopathologically, GC is subdivided into two subtypes, diffuse-type GC and intestinal-type GC, according to the Laurén classification [6]. The intestinal-type GC displays well-differentiated tubular or glandular structures, while the diffuse-type GC contains infiltrating neoplastic cells and undifferentiated or poorly-differentiated glandular structures. The two subtypes may also differ in clinical parameters, as patients with diffuse-type GC commonly show a higher recurrence rate and poorer patient prognosis compared to those with intestinal-type GC [7-9]. Despite of histological difference, these two subtypes pose different molecular profiles, such as E-cadherin expression [10, 11].

The repression of E-cadherin expression, a ‘master programmer’ of Epithelial Mesenchymal Transition (EMT), is a critical event in tumor invasion [12]. E-cadherin is a transmembrane protein crucial for maintaining intercellular homophilic adhesion of epithelial cells. It is responsible for cell adhesion and inhibits Wnt/β-catenin-mediated gene transcription, thereby maintaining cells in a cohesive, non-motile state [5]. The loss of E-cadherin expression is directly linked to loss of intercellular adhesion and is associated with enhanced invasiveness [13-15], and is therefore an indicator of poor prognosis for various cancers, including gastric cancer [16]. Unlike the intestinal-type GC, diffuse-type GC is associated with loss of expression or function of E-cadherin, partly attributable to genomic and epigenetic alterations such as inactivating germline and somatic mutations in *CDH1* [17], loss of heterozygosity and promoter hypermethylation [10, 13]. E-cadherin expression can also be repressed by various dysregulated signal transduction events in both GC subtypes during malignant progression as part of the EMT program, which activates E-cadherin transcriptional repressors [12]. In contrast to mechanisms for the genetic aberration of CDH1, the non-genetic molecular mechanisms of E-cadherin repression are much less characterized in GC.

Activation of the HGF-MET signaling pathway promotes cell scattering in cancer, and modulates other cellular behaviors such as cell invasion, motility, proliferation and cell survival [18-20]. The HGF-MET signaling is especially relevant in GC which harbors a high incidence of MET gene amplification and/or protein overexpression [19, 21-24]. HGF together with its receptor MET, triggers oncogenic signaling events which result in the mesenchymal transformation of tumor cells, resulting in attributes which promote tumor spread, including cell-scattering and invasion. HGF-MET effector pathways, including PI3K [25] and MAPK [14, 26], have also been implicated in E-cadherin repression and cell scattering in various carcinomas. Interestingly, there are evidences suggesting the involvement of actin-regulating factors in the HGF-MET pathway. It has been reported that villin, one of the gelsolin superfamily member, enhances HGF-induced motility and morphogenesis of EMT [27]. However, whether the gelsolin family members could alter E-cadherin to modulate cell motility and scattering in response to HGF is currently unknown. In this report we describe a novel role of gelsolin, an actin-modulating cytoskeletal protein and the founding member of gelsolin superfamily, in repression of E-cadherin expression through the HGF-MET pathway.

Gelsolin is required for cytoskeletal turnover through its actin-severing and capping activities. By virtue of these properties, combined with the ability to regulate protease secretion, gelsolin promotes cell invasion and migration in various carcinoma cell types [28-32]. It is currently unclear whether gelsolin confers similar properties in GC. Furthermore, in contrast to its role in invasion and migration, the role of gelsolin in intercellular adhesion is not well studied. Gelsolin was previously reported to interfere with intercellular adhesion in canine kidney cells [29] and also in the regulation of β1-integrin affinity and cell adhesion in leukemic cells [33]. In this study we showed that gelsolin inhibits intercellular adhesion in GC cells by regulating the expression of E-cadherin. We also determined that gelsolin promoted GC cell scattering in response to HGF *via* the PI3K-Akt pathway. Our findings reveal a novel function of gelsolin in the mediation of HGF-induced PI3K/Akt activation, which leads to E-cadherin repression and scattering of GC cells. Hence, gelsolin functions as an important pro-disseminative protein in GC cells.

## RESULTS

### Gelsolin expression is increased in diffuse-type compared to intestinal-type gastric cancers

**Figure 1:**
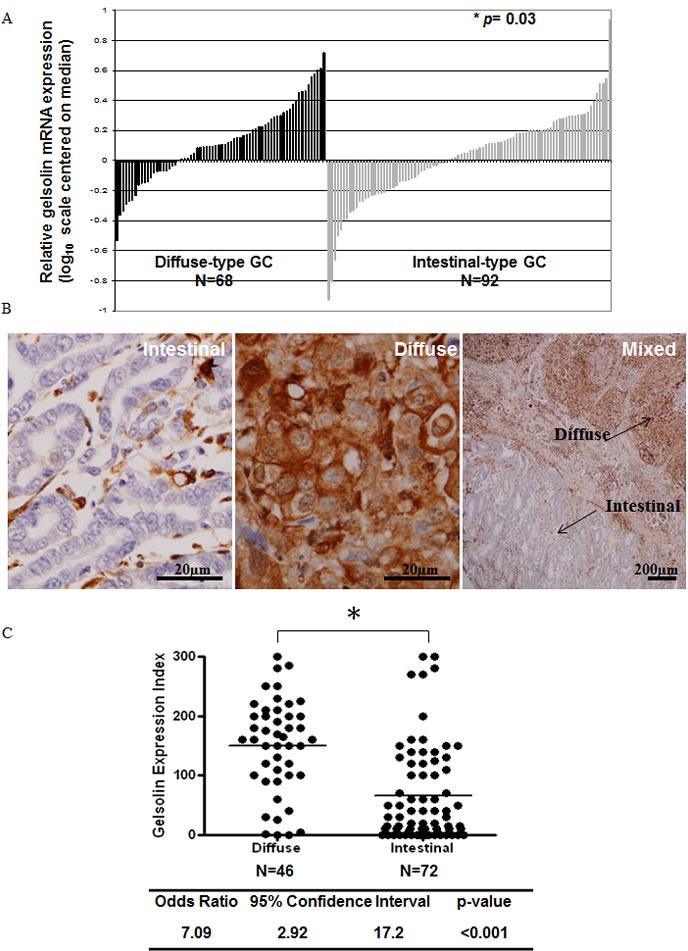
Increased Gelsolin expression in diffuse-type gastric cancer **A.** Relative gelsolin gene expression in diffuse-type and intestinal-type gastric cancer. *N* = 68 (Diffuse-type), *N* = 92 (Intestinal-type). **B.** IHC staining of gelsolin expression in intestinal, diffuse and mixed gastric cancer tissues. **C.** Gelsolin expression index in diffuse and intestinal type gastric cancers. *N* = 46 (Diffuse-type), *N* = 72 (Intestinal-type). Score was calculated by the product of staining intensity and corresponding % positivity, where intensity ranges from 0 (no observable staining) to 3 (intense staining). Paired *T*-test was used to compute *p*-value shown.

We first examined the expression of gelsolin and E-cadherin in human GC samples by microarray analysis and/or immunohistochemistry (IHC). Microarray analysis was conducted on mRNA from 160 gastric tumors, of which 68 samples were classified under diffuse-type and 92 under intestinal-type GC based on Lauren's classification. The comparison between the 2 GC subtypes showed higher gelsolin mRNA expression in diffuse-type GCs (*p* = 0.03), based on unpaired student's *t*-test analysis (Figure 1A). Gelsolin expression was further investigated by IHC in 118 parrafin-embedded primary gastric tumors (matched with adjacent normal tissues) comprising 46 diffuse-type and 72 intestinal-type gastric tumors. High expression of gelsolin was observed in the muscularis propria as previously reported [34, 35]. In normal gastric mucosa, gelsolin staining exhibited a heterogenous and gradient pattern, where the surface mucosal cells expressed high levels of gelsolin and the deeper glands showed undetectable to low expression (Suppl. Figure 1A). Interestingly, whilst the majority of intestinal-type GCs stained poorly for gelsolin, diffuse-type tumors expressed higher gelsolin levels (Figure 1B). In a small proportion of tumors, mixed features comprising both diffuse and intestinal-type tumor tissues within the same tumor were observed. In these ‘mixed-type GCs’ the diffuse component also showed more intense gelsolin staining compared to the intestinal component (Figure 1B). Gelsolin expression in diffuse-type GCs was significantly increased above that observed in intestinal-type GCs, as reflected in the Gelsolin Expression Index which was calculated based on IHC staining intensities (*p* = 0.0015, Unpaired *t*-test) (Figure 1C). Overall, the observations of high gelsolin expression in GC tumors displaying diffusely infiltrative phenotype and low gelsolin expression in GC tumors with cohesive morphology are indicative of a positive association between gelsolin and gastric cancer invasiveness.

### Gelsolin is increased in intestinal-type GC metastatic to lymph nodes

Recently we reported that gelsolin expression was increased in the liver metastases of a subset of colon cancer patients [28]. We sought to investigate if a similar correlation exists in the intestinal-type primary tumors and their metastases, since the latter generally expresses relatively low gelsolin levels. 20 intestinal-type lymph node metastases were examined for gelsolin expression by IHC. The majority of lymph node metastases (17 of 20 tumour pairs) expressed significantly higher gelsolin levels compared to their corresponding primary tumors (*p* = 0.004, Paired *t*-test) (Supp. Figure 1B-1D). Our data suggests that increased gelsolin expression may also favour the development of metastases in intestinal-type GC, and is consistent with the reported pro-invasive function of gelsolin [28, 29].

### Expression of gelsolin inversely correlates with wild-type E-cadherin

**Figure 2:**
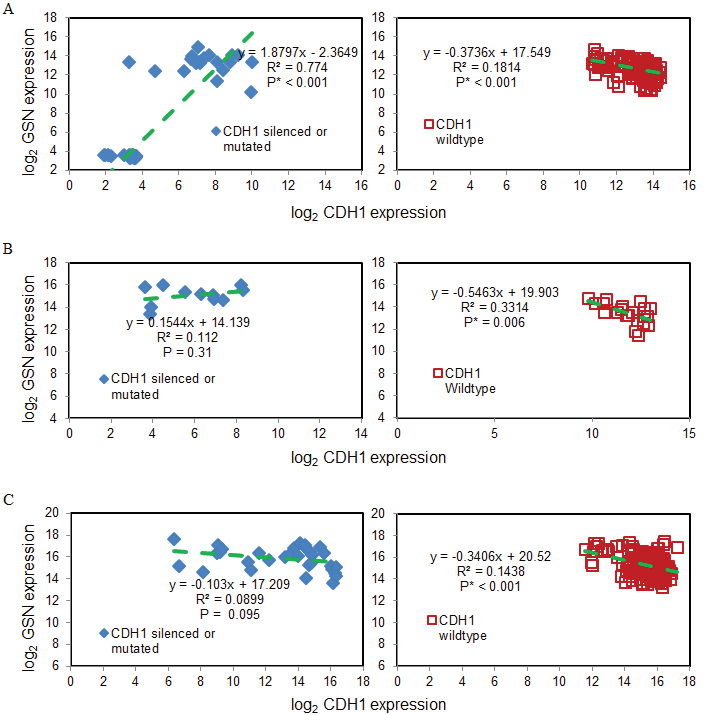
Gelsolin expression inversely correlates with wild-type E-cadherin expression **A.** GSE15460 (75 samples with silenced or mutated CDH1 & 181 samples of wild-type CDH1), **B.** GSE65801 (11 samples with silenced or mutated CDH1 & 21 samples of wild-type CDH1) and **C.** TCGA STAD (32 samples with silenced or mutated CDH1 & 186 samples of wild-type CDH1) for silenced or mutated CDH1 (left) and wildtype CDH1 (right). Green dashed lines are the linear regression results.

E-cadherin, an important protein encoded by CDH1 gene to mediate cell adhersion, has been reported to be commonly mutated in diffuse-type gastric cancer to contribute to cancer dissemination (6,35-37). We examined the association of gelsolin and E-cadherin expression. Three gastric cancer cohorts from GEO and TCGA were analysed (Figure 2). There was a significant, negative correlation between gelsolin and CDH1 for patient samples with wild-type CDH1 across all three cohorts. This correlation was not found for patient samples with silenced or mutated CDH1. Therefore, the expression of gelsolin inversely correlates with wild-type E-cadherin but not with its mutated form. Our data suggests that gelsolin may be involved in regulating functional E-cadherin expression.

### Loss of gelsolin abrogates invasion of gastric cancer cells and promotes E-cadherin-dependent intercellular adhesion of gastric cancer cells

Since diffuse GC tissues and metastatic GC cell lines revealed a possible correlation between gelsolin and tumor progression and invasiveness, we examined the effect of gelsolin on invasion in two gastric cancer cell lines with high gelsolin expression, MKN28 and AGS. We used siRNA knockdown of gelsolin to decrease protein expression in both cell lines by > 95% (Supp. Figure 2A), and this corresponded with a significant reduction in invasive capacity through matrigel in response to a serum-concentration gradient (Supp. Figure 2B). There were no changes in cell proliferation or cell death when gelsolin levels were reduced by siRNA transfection (Supp. Figure 2C-2D). These findings provide evidence that gelsolin is important for the invasiveness of gastric cancer cells, consistent with previous reports on other tumor types [28, 29].

**Figure 3:**
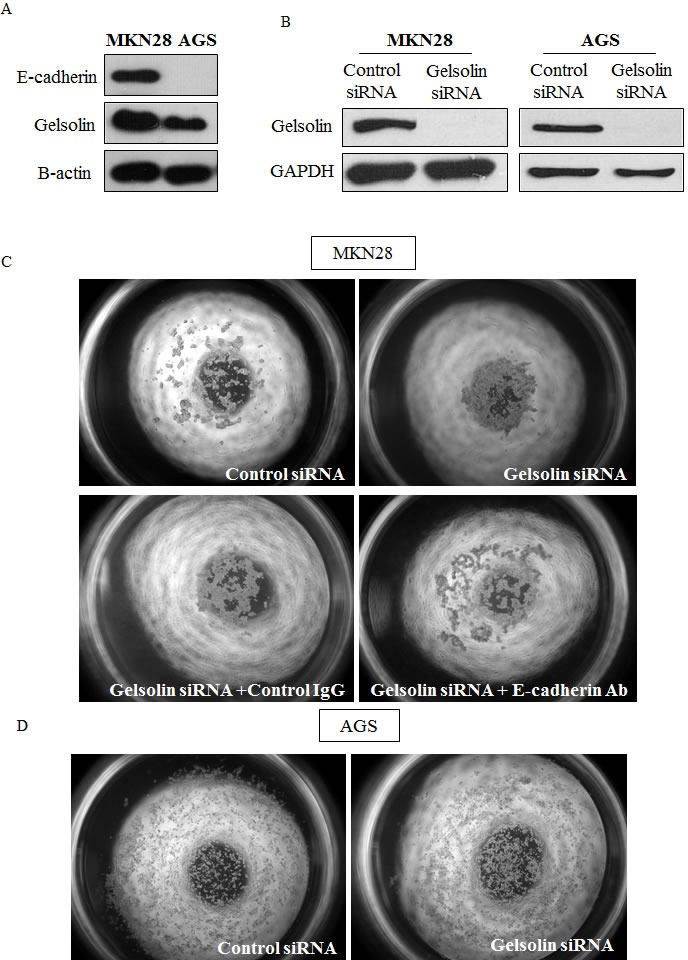
Loss of Gelsolin promotes E-cadherin-dependent intercellular adhesion of gastric cancer cells **A.** Western blot of basal E-cadherin and Gelsolin protein levels in MKN28 and AGS cells. **B.** MKN28 and AGS cells were transfected with control scrambled RNA or siGelsolin RNA. Western blot was conducted after 48h to check efficiency of knockdown. **C.** Cell aggregation assay on soft agar was conducted with MKN28 cells transfected with control scrambled RNA or siGelsolin RNA. Cells were also incubated with function-blocking E-Cadherin antibodies. Images are representatives from three independent experiments. **D.** Cell aggregation assay was conducted with AGS cells transfected with control scrambled RNA or siGelsolin RNA. Images are representatives from three independent experiments.

The loss or reduction of intercellular adhesion is a hallmark of malignancy which is closely associated with a propensity for invasion and distant metastasis [13]. To determine the effect of gelsolin on intercellular adhesion, cell aggregation assays were conducted by culturing GC cells in soft agar for 24 hours. The semi-solid substrate prevents the adherence of cells and reduces cell movement, thereby allowing the relative assessment of the strength of intercellular adhesion (De Corte et al., 2002). As E-cadherin is a prominent epithelial cell adhesion molecule mediating tight intercellular adhesion, it is an important mediator of cellular aggregation. We thus performed the cell aggregation experiments using E-cadherin-positive MKN28 and E-cadherin-deficient AGS cells as a negative control (Figure 3A). Western blot was used to confirm the knockdown efficiency of gelsolin siRNA (Figure 3B). We observed that whilst control siRNA-transfected MKN28 cells were loosely scattered in small clusters, gelsolin siRNA-transfected cells formed large aggregated cell clusters, indicating that the loss of gelsolin enhanced intercellular adhesion of MKN28 cells (Figure 3C). Furthermore, we observed that the cellular aggregation of MKN28 cells induced by gelsolin siRNA was abrogated by treatment of cells with function-blocking E-cadherin antibodies, where cells then assumed loose aggregation patterns similar to the control siRNA-transfected cells (Figure 3C). Hence, the cellular aggregation resultant upon the loss of gelsolin is potentially associated with enhanced E-cadherin function, without which cells consequently become loosely scattered. In contrast, gelsolin depletion in E-cadherin-negative AGS cells did not affect cellular aggregation (Figure 3D). Our data therefore suggests that gelsolin only influences infiltrative behavior of GC cells with functional E-cadherin.

### Loss of gelsolin increases E-cadherin expression in gastric cancer cells

**Figure 4:**
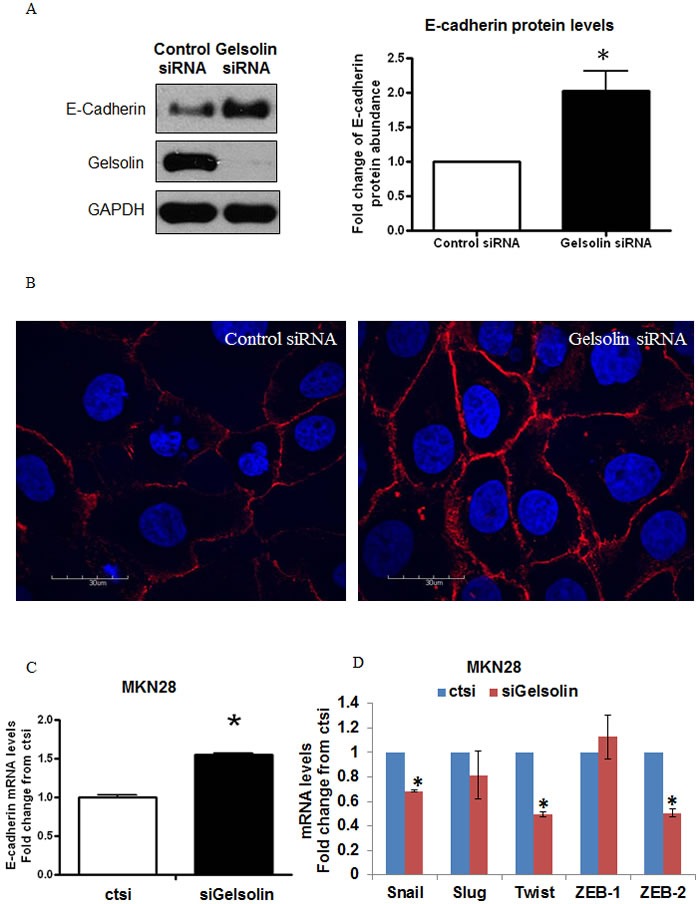
Loss of Gelsolin increases E-Cadherin expression in gastric cancer cells **A.** MKN28 cells were transfected with control scrambled RNA or siGelsolin for 48h. Left: Western blot of E-Cadherin expression levels after transfection. Right: Densitometric analysis of E-Cadherin protein levels normalized to GAPDH protein levels after transfection. Values represent mean ± SD, *n* = 3, *P < 0.05 *vs*. control. **B.** Immunofluorescence staining of E-Cadherin expression levels in MKN28 cells after transfection with control scrambled RNA or siGelsolin for 48h. Images are representatives from three independent experiments.**C.** E-Cadherin mRNA levels in MKN28 cells after transfection with control scrambled RNA or siGelsolin for 48h, normalised to GAPDH mRNA levels. Values represent mean ± SD, *n* = 3, *P < 0.05 *vs*. control. **D.** Snail, Slug, Twist, ZEB-1 and ZEB-2 mRNA levels in MKN28 cells after transfection with control scrambled RNA or siGelsolin for 48h, normalised to GAPDH mRNA levels. Values represent mean ± SD, *n* = 3, *P < 0.05 *vs*. control.

The loss of intercellular adhesion is frequently attributed to the downregulation and/or dyslocalization of cell adhesion molecules such as E-cadherin [36, 37]. We observed that siRNA mediated depletion of gelsolin in GC cells increased the protein levels of E-cadherin by more than 2 folds in MKN28 cells (Figure 4A). The increase in E-cadherin by gelsolin knockdown was confirmed by using two siRNA targeting gelsolin (Supp Figure 3). Similarly, knockdown of gelsolin induced the protein expression of E-Cadherin in MKN7 and MKN74 cells (Supp Figure 4A & 4B), supporting a role of gelsolin in regulating E-Cadherin expression. Concomitantly, E-cadherin expression at intercellular junctions based on immunofluorescence detection was increased in the gelsolin-depleted cells compared to control siRNA-transfected cells (Figure 4B). These results corroborate the cellular aggregation data and suggest that loss of gelsolin in GC cells promotes increased E-cadherin expression and its localization to intercellular junctions, resulting in an increased ability of the cancer cells to aggregate.

We next assessed whether the gelsolin-mediated E-cadherin downregulation is also observed at the transcriptional level. We observed that siRNA depletion of gelsolin significantly increased E-cadherin expression at the mRNA level, using real-time PCR. To determine if depletion of gelsolin increased E-cadherin expression *via* transcriptional activation, we examined the effect of gelsolin depletion on the expression of well-known E-cadherin transcriptional repressors, namely Snail, Slug, Twist1, ZEB-1 and ZEB-2. We observed that gelsolin depletion reduced the mRNA levels of Snail, Twist and ZEB-2, concordant with an increase in E-cadherin mRNA expression upon gelsolin depletion in both MKN28 and MKN74 cells (Figure 4C-4D, Supp. Figure 4C). Hence, gelsolin may be a novel repressor of E-cadherin expression by modulating the expression of Snail, Twist and ZEB-2.

### Gelsolin is induced by hepatocyte growth factor (HGF) and is essential for HGF-induced E-cadherin downregulation and cell scattering

We have thus far observed that gelsolin is an important repressor of E-cadherin expression in GC. The activation of HGF-MET signaling is a well-established stimulus leading to decreased expression of E-cadherin and cell scattering in GC cells [38]. We therefore proceeded to determine whether gelsolin mediates HGF-induced E-cadherin repression. For these experiments, we utilized MKN28, MKN74, and TMK-1 GC cell lines, all of which express c-MET and are able to respond to HGF-induced growth activation.

**Figure 5:**
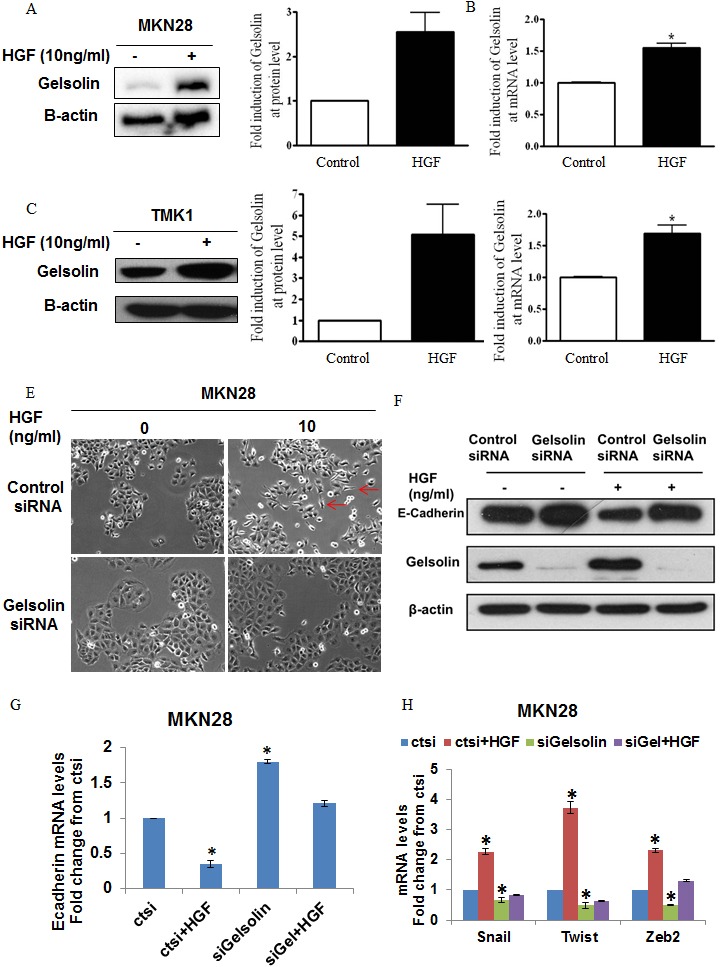
Gelsolin mediates HGF-induced E-cadherin downregulation and cell scattering of gastric cancer cells **A.** Left: Western blot analysis of gelsolin levels in MKN28 cells treated with HGF for 48h. Right: Densitometric analysis of gelsolin protein levels in MKN28 cells treated with HGF for 48h. Values represent mean ± SD, *n* = 3, *P < 0.05 *vs*. control. **B.** Gelsolin mRNA levels in MKN28 cells treated with HGF for 48h, normalized against GAPDH levels. Values represent mean ± SD, *n* = 3, *P < 0.05 *vs*. control. **C.** Left: Western blot analysis of gelsolin levels in TMK1 cells treated with HGF for 48h. Right: Densitometric analysis of gelsolin protein levels in TMK1cells treated with HGF for 48h. Values represent mean ± SD, *n* = 3, *P < 0.05 *vs*. control. **D.** Gelsolin mRNA levels in TMK1cells treated with HGF for 48h, normalized against GAPDH levels. Values represent mean ± SD, *n* = 3, *P < 0.05 *vs*. control. **E.** Light microscopy images of MKN28 cells transfected with ctsi or siGelsolin and treated with HGF for 48h (x100 magnification). Images are representatives from three independent experiments. **F.** Western blot of MKN28 cells transfected with ctsi or siGelsolin and treated with HGF for 48h and blotted for E-Cadherin protein expression. **G.** E-cadherin mRNA levels in MKN28 cells transfected with ctsi or siGelsolin and treated with HGF for 48h, normalized against GAPDH mRNA levels. Fold changes of mRNA compared to ctsi transfected cells were presented. Values represent mean ± SD, *n* = 3, *P < 0.05 *vs*. control. **H.** Snail, Twist and Zeb2 mRNA levels in MKN28 cells transfected with ctsi or siGelsolin and treated with HGF for 48h, normalized against GAPDH mRNA levels. Fold changes of mRNA compared to ctsi transfected cells without HGF treatment were presented. Values represent mean ± SD, *n* = 3, *P < 0.05 *vs*. control.

We observed that expression of gelsolin mRNA and protein were increased following treatment with HGF in MKN28, MKN74, and TMK1 cells (Figure 5A-5D, Supp. Figure 6A), an association that was hitherto unreported. Expectedly, HGF promoted partial loss of intercellular adhesion and a mesenchymal cell phenotype in the three cell lines (Figure 4E, Supp. Figure 5A, and Supp. Figure 6B). Notably, the reduction in intercellular adhesion was more distinct in MKN28 and MKN74, which normally exhibit an epithelial morphology compared to TMK1, which assumes a slightly scattered appearance in culture. HGF-induced phenotypic changes in MKN28, MKN74 and TMK1 cells were abrogated by siRNA reduction of gelsolin levels, indicating that gelsolin is necessary for HGF to promote GC cell scattering (Figure 4E, Supp. Figure 5A, and Supp. Figure 6B). HGF stimulation repressed E-cadherin protein, consistent with the reported effects of HGF, and this reduction was abrogated by gelsolin depletion (Figure 5F, Supp. Figure 5B, and Supp. Figure 6C), indicating that gelsolin modulates the HGF-induced repression of E-cadherin expression. Importantly, the levels of gelsolin and E-cadherin correlated with the morphological changes observed, further confirming the role of gelsolin in HGF-induced cell scattering. Furthermore, depletion of gelsolin impaired the cellular response to HGF-induced gene transcriptional changes, which includes repression of E-cadherin and a concomitant increase in the mRNA expression levels of Snail, Twist and ZEB-2 (Figure 5G and Supp. Figure 5C). The effect of gelsolin knockdown on HGF-induced transcription was also observed in MKN74 cells, where HGF-induced expressions of Snail, Twist, ZEB-1 and ZEB-2 were significantly inhibited by silencing gelsolin (Supp. Figure 6D-6E). Taken together our data indicates that gelsolin is a downstream effector of HGF-induced E-cadherin repression and cell scattering.

### Gelsolin mediates the HGF-induced repression of E-cadherin *via* PI3K-Akt pathway

**Figure 6:**
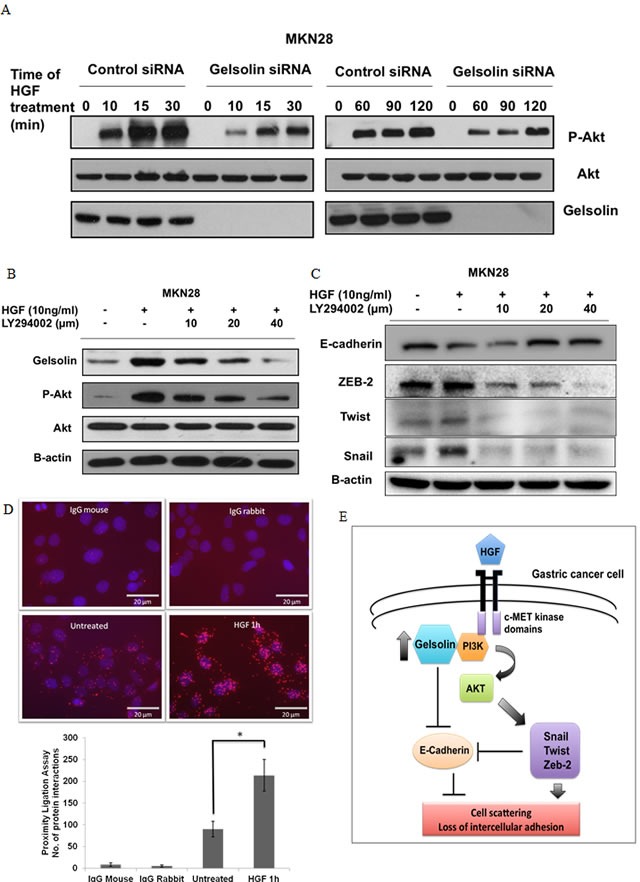
Gelsolin mediates HGF-induced E-cadherin downregulation *via* PI3K-Akt pathway **A.** Western blot of MKN28 cells transfected with ctsi or siGelsolin before treatment with HGF for 0-120 min and analyzed for gelsolin, phosphorylated Akt and Akt protein levels. **B.** Western blot of MKN28 cells pre-incubated with 10, 20 and 40μM LY294002 for 1h before treatment with HGF for 48h and analyzed for gelsolin, phosphorylated Akt and Akt protein levels.**C.** Western blot of MKN28 cells treated as in **A.** and analyzed for protein levels of EMT markers E-Cadherin, Zeb2, Twist and Snail. **D.** Proximity ligation assay of MKN28 cells treated with 10ng/ml HGF for 1h and stained with PI3K and Gelsolin antibodies. Top: Microscopy images of cells. Red spots are representative of the interactions between PI3K and Gelsolin, each red spot is equivalent to one molecular interaction. Nuclei are stained with DAPI. Images were acquired using a fluorescence microscope at x400 magnification. Scale Bar = 20μm. Bottom: Quantitative analysis of number of molecular interactions counted from 3 representative images taken at random fields from 2 experiments. Values represent mean ± SD, *n* = 2, *P < 0.05 *vs*. untreated. **E.** Model of HGF-induced cell scattering and loss of intercellular adhesion in gastric cancer cells involving gelsolin-PI3K-Akt pathway.

As the binding of HGF to c-MET receptors initiates multiple intracellular signaling events, we sought to delineate the specific downstream pathway(s) mediating E-cadherin repression in MKN28 and TMK-1 cells, including the PI3K-Akt and MEK-MAPK pathways which have been previously implicated in the downregulation of E-cadherin [14, 26, 39]. Cells were serum-starved for 24 hours before treatment with 10ng/mL of HGF. Western blot analysis showed that HGF treatment resulted in activation of the PI3K-Akt pathway, as evident from the increased phosphorylated Akt (pAkt), which was sustained for at least 120 minutes after HGF stimulation (Figure 6A and Supp. Figure 7A). To determine whether gelsolin modulated this HGF-stimulated signaling, cells were depleted of gelsolin by use of siRNA, followed by serum-starvation and stimulation with HGF. As shown in Figure 5A and Supp. Figure 4A, gelsolin siRNA depletion resulted in inhibition of Akt phosphorylation in both MKN28 and TMK1, indicative that gelsolin is a modulator of the HGF-induced PI3K-Akt pathway in GC cells.

To determine if the increased expression of gelsolin stimulated by HGF is dependent on PI3K, LY294002 was used as a specific inhibitor of PI3K. The increase in gelsolin expression upon HGF stimulation was abrogated by dose-independent increases in LY294002. Phosphorylation of Akt was used as a measure of the inhibitory effect of LY294002, where increasing doses inhibited the levels of p-Akt (Figure 6B). Simultaneously, HGF treatment resulted in a repression of E-cadherin corresponding with an upregulation of E-cadherin transcriptional repressors Snail, Twist and ZEB-2 in MKN28 and TMK-1 cells. These changes in gene expressions were abrogated by LY294002, indicating that the PI3K-Akt signaling pathway mediated HGF-dependent E-cadherin repression (Figure 6C).

It was reported previously that gelsolin interacts with PI3K in a complex in osteoclast podosomes [40] and stimulates PI3K activity [41]. Using a proximity ligation assay which detects protein interactions in cells *in situ*, we observed an increased association between gelsolin and PI3K in MKN28 cells upon HGF treatment (Figure 6D). Whether this physical interaction is required for PI3K activity (and therefore the activation of PI3K-Akt pathway) is of interest for future studies. Taken together our findings strongly suggest that gelsolin promotes the loss of E-cadherin by functioning as a mediator of the HGF-dependent PI3K-Akt pathway, thus regulating cell scattering and intercellular adhesion (Figure 6E).

## DISCUSSION

During the course of dissemination, epithelial cells may undergo the EMT program, during which closely-associated, non-invasive cells transform into loosely-associated, migratory and invasive cells. E-cadherin expression is frequently lost or reduced during this phenotypic transition [12, 38]. Lower E-cadherin levels were observed in the diffuse-type GC compared to intestinal-type GC, which correlated with the morphological characteristics and consistent with the varying aggressiveness of these two subtypes [10, 42]. Somatic alterations in E-Cadherin have been found in approximately 30% of GC, with frequencies ranging from 3-50% in sporadic diffuse-type GC [43, 44]. Therefore, besides genetic aberrations leading to downregulation of E-cadherin, non-genetic molecular mechanisms contributing to loss of E-cadherin function have become of increasing importance. In this study we show that the cytoskeletal protein, gelsolin, is an important determinant of GC invasion and cell scattering by mediating E-cadherin repression *via* the HGF-PI3K-Akt signaling pathway.

Gelsolin was found to be upregulated in diffuse-type GC compared to intestinal-type GC, indicating that gelsolin is associated with a more infiltrative phenotype. Interestingly, we also observed a differential pattern of gelsolin expression in the intestinal-type primary tumors expressing low gelsolin. It was found that gelsolin expression was higher in a subset of lymph node metastases compared to their primary intestinal tumors. The evidence is also concordant with data showing higher gelsolin levels in cell lines derived from metastatic sites, as well as a previous reports documenting increased gelsolin expression in tumor metastases [28, 45]. It is currently unclear whether gelsolin has distinct functions in the pathogenesis of these two subtypes at different stages, especially with respect to their disseminative potential.

Our initial *in vitro* data revealed a decrease in invasiveness and loss of cellular aggregation upon gelsolin depletion in GC cells. This is corroborated by previous reports that gelsolin is required for tumor cell invasion and cellular aggregation in various carcinomas [33, 46, 47]. Since the effect of gelsolin on cellular aggregation is determinant upon the presence of functional E-cadherin, we investigated the possible effects of gelsolin on E-cadherin expression. We observed that there was an inverse correlation of gelsolin with E-cadherin expression, where depletion of gelsolin led to increases in E-cadherin expression, therefore contributing to enhanced cellular aggregation and perturbed cell scattering properties. This is accompanied by decreases in E-cadherin transcriptional repressors Snail, Twist and Zeb-2. Perturbations of E-cadherin following gelsolin overexpression has been previously reported in cardiomyocytes [48], supporting our inverse correlation between gelsolin and E-cadherin. Besides functioning as actin-binding proteins, nuclear roles of gelsolin in regulation of transcription have been reported. Gelsolin and its family members such as Flightless-I (FliI), have been shown to act as transcriptional coactivators of nuclear receptors such as estrogen receptor, androgen receptor and glucocorticoid receptor [49], and FliI was found to alter transcription of genes involved in β-catenin signaling such as cyclin D1 [50]. These provide support for gelsolin as a possible transcriptional regulator of E-cadherin. However, the exact mechanism of how gelsolin inhibits nuclear transcription of E-cadherin remains to be elucidated.

We also found that HGF activates the PI3K-Akt pathway in GC cells, leading to induction of E-cadherin repressors Snail, Twist and ZEB-2, and concomitant downregulation of E-cadherin. Silencing of gelsolin blocked the upregulation of E-cadherin transcriptional repressors Snail, Twist and Zeb2, and the repression of E-cadherin in response to HGF, showing the critical role of gelsolin in cellular response to c-MET activation. Interestingly, siRNA depletion of gelsolin resulted in a stark inhibition of Akt phosphorylation in response to HGF, indicating that gelsolin expression is pivotal to the activation of PI3K-Akt pathway under HGF-MET signaling transduction event. In contrast, little or no p-Akt was detected in TMK-1 cell line following 2 hours of HGF treatment, corresponding with a sustained effect of gelsolin siRNA. The delay in gelsolin induction in response to HGF in TMK-1 may be attributed to its relatively low expression of MET [51], in contrast to MKN28 which expresses higher levels of c-MET [52].

Activation of HGF signaling also led to an induction of gelsolin, a phenomenon that decreased upon inhibition of PI3K, suggesting that gelsolin expression may be regulated by PI3K. Restoration of E-cadherin levels were also observed, which is consistent with an earlier report [25]. The reduction of gelsolin and restoration of E-cadherin following PI3K inhibition blocked the induction of E-cadherin transcriptional repressors upon HGF treatment, suggesting that gelsolin-PI3K-Akt signaling could be involved in regulating the EMT transcription factors. A previous study reported that gelsolin inhibits E-cadherin-mediated cell aggregation in transformed canine cells, but the authors found no effect of gelsolin on the integrity of E-cadherin-catenin adherens complex [29]. Consistent with our findings, an earlier study showed that inhibition of PI3K represses gelsolin protein levels and decreased migration and invasion of hepatocarcinoma cells, providing further evidence for gelsolin's involvement in the PI3K-Akt pathway [53]. This finding also strongly supports an oncogenic role for gelsolin, since the PI3K-Akt axis is a major downstream pathway that is activated by oncogenic signaling mediated by several other receptor tyrosine kinases besides c-MET, such as EGFR and ERBBs [54].

Gelsolin has been shown to physically associate with PI3K [40, 55] and promote its activity [41]. Interestingly we observed a greater degree of interaction between gelsolin and PI3K in the presence of HGF, which correlated with higher Akt phosphorylation. Further studies are warranted to confirm whether gelsolin influences Akt phosphorylation through its physical association with PI3K, and whether gelsolin exerts similar effects on PI3K activation mediated by other growth factor receptor signal transduction events.

In summary, our findings have shown that gelsolin confers disseminative properties in GC cells, by promoting cell invasion as well as functioning as a determinant in the HGF-PI3K-Akt signaling pathway which mediates E-cadherin downregulation and cell scattering. Although further molecular insights of these processes still await to be elucidated, our study have shed more light on the oncogenic functions of gelsolin involved in driving GC dissemination. Combined with the clinical data showing higher gelsolin levels in diffuse and metastatic GC, a novel role for gelsolin as a potential biomarker in disease diagnosis and target for therapy is unraveled.

## MATERIALS AND METHODS

### Human samples

Primary gastric tumors were obtained from National Cancer Centre, Singapore with approvals from Research Ethics Review Committee, and signed patient informed consent. Total RNA was extracted using Qiagen RNA extraction reagents (Qiagen, Venlo, Limburg, Netherlands) according to the instructions of the manufacturer and hybridized to Affymetrix Human Genome U133 plus Genechips (HG-U133 Plus 2.0, Affymetrix, Santa Clara, CA, USA). Raw data obtained after chip-scanning was pre-processed using the MAS5 algorithm (Affymetrix). Data were subjected to Log 10 transformation followed by median-centered across all probe sets for each sample (primary tumor). The centering is such that the median of expression in each sample is zero. 160 were classified by pathological diagnosis into diffuse-type GCs (68 cases) and intestinal-type (92 cases).

Formalin-fixed and paraffin-embedded (FFPE) primary tumor and matched normal tissue samples from 118 patients with gastric cancer were obtained from the Department of Pathology at the National University Hospital System, Singapore, under an institutionally approved protocol.

### Immunohistochemistry and scoring methods

Briefly, 5μm thick tumor sections were deparaffinized, boiled in Antigen Unmasking Solution (Vector Laboratories, Inc Burlingame, CA, USA) at 95°C for 10 minutes and blocked with hydrogen peroxide for 5 minutes before primary antibody incubation overnight at 4°C. Both primary anti-gelsolin (Abcam, Cambridge, England, UK) and anti-E-cadherin (Millipore, Darmstadt, Germany) monoclonal antibodies were used at a dilution of 1:500. The antibody used was previously determined to be specific for gelsolin by western blots of protein lysates from several human cell lines, as well as cell lines transfected with a gelsolin-expressing plasmid. All other steps were performed using the avidin-biotinylated horseradish peroxidase complex method (Envision+, DAKO, Glostrup, Denmark). Samples were incubated with DAB+ chromogen (DAKO, Glostrup, Denmark) for 3 minutes and subsequently counterstained with hematoxylin. Gelsolin expressions was quantified as described previously [56, 57]. The staining score was expressed as the Gelsolin Expression Index ( = intensity X corresponding % positivity), where intensity ranges from 0 (no observable staining) to 3 (intense staining), and % positivity ranges from 0-100%. The normal epithelial cells adjacent to tumor cells (if available) were used as scoring references. Paired *T*-test was used for statistical analysis.

### Bioinformatical analysis of gelsolin and E-cadherin expression

Three gastric cancer cohorts were taken from GEO database (http://www.ncbi.nlm.nih.gov/gds) and TCGA (https://tcga-data.nci.nih.gov/tcga/). The two GEO datasets with the GEO accession numbers of GSE15460 and GSE65801 [58] were generated using microarray technologies, while the third cohort was the RNA-seq data of the TCGA Stomach Adenocarcinoma (STAD) set. The raw CEL files were downloaded for the two microarray datasets, and the gene expression intensities were extracted by RMA and further normalized by the Cross-Correlation method [59]. For TCGA RNA-seq data, raw counts of Level 3 were downloaded and only non-intestinal gastric adenocarcinoma including diffuse-type were used as the third cohort. The RNA-seq counts were normalized using the total numbers of mappable reads across all samples. All normalized data were then log2-transformed, and normalized log2 transformed expression data for GSN and CDH1 were subsequently used for the expression correlation study.

### Cell lines

MKN7, MKN28, MKN74, TMK1, AGS, are commercially-available cell lines from ATCC, Manassas, VA, USA.

### Cell culture and drug treatment

Cell lines MKN7, MKN 28, MKN74 and AGS were cultured in RPMI-1640 (Sigma, St. Louis, MO, USA) and TMK1 in DMEM, supplemented with 10% fetal bovine serum (FBS; Gibco, Grand Island, NY, USA) and 1% penicillin streptomycin (Gibco). All cell lines are maintained in a humidified 37°C incubator with 5% CO_2_. Cells were plated at similar density prior to treatments. Human recombinant hepatocyte growth factor (HGF) was purchased from Sigma while PI3K inhibitor LY294002 and was purchased from Promega, Madison, WI, USA.

### Cellular aggregation

Aggregation assays were performed as described previously [29]. After 24 hours of siRNA transfection, cells were detached and seeded on top of a semi-solid agar coated 96-well plate in the presence or absence of 25μg/ml of E-cadherin neutralizing monoclonal antibody (Millipore). Aggregation formation was evaluated after 24 hours under an inverted phase-contrast microscope (ZEISS, Jena, Germany).

### Invasion assay

BD Matrigel basement membrane matrix (BD Biosciences, Franklin Lakes, NJ, USA) was diluted and coated onto the upper chamber of transwells (Corning Costar, Tewksbury MA, USA) according to manufacturer's instructions. Cells were transfected with siRNA duplex for 24 hours, then cultured in serum-free medium for 24 hours. Next, they were seeded onto the upper chamber of the matrigel-coated transwell containing serum-free medium. Complete medium with 10% FBS was added to the lower chamber. Cells were allowed to migrate at 37°C in a humidified incubated supplemented with 5% CO_2_ for 24 hours. Cells in the upper chamber were then removed and the upper side of the membrane thoroughly cleaned. Invading cells were fixed and stained with either crystal violet or propidium iodide. Invaded cells stained with propidium iodide were counted using the inverted fluorescent microscope in at least 10 representative fields.

### Western blot analysis

Cells were harvested and protein extracted using RIPA lysis buffer with protease inhibitor cocktail mix (Roche Diagnostics, Mannheim, Germany). Proteins were separated by electrophoresis under denaturing and reducing conditions. Monoclonal antibodies against gelsolin (Abcam), E-cadherin (Millipore), AKT and phosphorylated Akt, Erk and phosphorylated Erk (Cell Signaling, Beverly, MA, USA) and GAPDH (Santa Cruz Biotechnology Inc, Dallas, TX, USA) were used.

### Cell proliferation ELISA

Cells were transfected with siRNA duplex for 24 hours, then re-seeded in a 96-well plate in the medium at a density of 1000 cells/well and left overnight to attach. The following day, BrdU were added to each well. Cells were fixed and stained after 24 hours according to the manufacturer's instructions (Cell Proliferation ELISA for BrdU; Roche, Basel, Switzerland). Colorimetric analysis was performed with an ELISA plate reader (Biochrom Asys UVM 340; Biochrom, Holliston, MA, USA).

### Cell death

Cells were transfected with siRNA duplex for 72 hours, and then trypsinized and resuspended in PBS, followed by fixation in ice-cold 70% ethanol. Cellular DNA was stained with propidium iodide solution (PBS containing 50mg/ml propidium iodide, 0.1% Triton X-100 and 100mg/ml RNaseA) and analyzed using FACS Vantage SE Flow Cytometry system (Becton Dickinson, NJ, USA).

### Gene silencing by RNA interference

Stealth siRNA targeting gelsolin was purchased from ThermoFisher Scientific (HSS104526 of siGel1 and HSS104524 of siGel2. siGel1 was used as gelsolin siRNA unless otherwise stated). 10nM of the Stealth siRNA, complexed with lipofectamine, was used to silence gelsolin expression in cells. Medium GC control siRNA which matched the GC content of the gelsolin siRNA used was included as controls. Cells were harvested at 72 hours and treated as used in other assays as described.

### Real-time PCR assay

Total RNA from cell lines were isolated from cultures of each experiment group with the Qiagen RNeasy kit as described by the manufacturer. 500-1000ng of cDNA was used for amplification, carried out on the 7500 Fast Real-Time PCR System. The thermal cycling conditions were as follows: one cycle at 95°C for 10 minutes, followed by 40-50 cycles of denaturation at 95°C for 15 seconds and annealing extension at 60°C for 1 minute. The specific primers used were designed by manufacturer to detect Gelsolin, E-cadherin, Snail, Slug, Twist, ZEB-1 and ZEB-2. Glyceraldehyde-3-phosphate (GAPDH) was including as an internal control. All reagents are from Applied Biosystems (Foster City, CA, USA).

### Proximity ligation assay

The mouse/rabbit red starter Duolink kit (Olink, Uppsala, Sweden) was used for this experiment. The cells were seeded on glass slides and treated with 10ng/ml HGF for one hour. Cells were then fixed with 4% paraformaldehyde for 15 minutes at room temperature and permeabilized with 0.25% Triton in PBS for 10 minutes at room temperature. After permeabilization the cells were incubated in the blocking buffer (provided with the kit) for 30 minutes at 37°C. Cells were then incubated with the primary antibodies against gelsolin (Abcam) and PI3K (Cell Signaling, Beverly, MA, USA) diluted in the antibody diluents for overnight at 4°C. On the next day, cells were washed in Buffer A 3 times for 15 minutes and incubated with the PLA probes for one hour at 37°C. This was followed by a 10 and 5 minutes wash in Buffer A. Ligation was carried out at 37°C for one hour followed by a 10 and 5 minutes wash in Buffer A. The cells were then incubated with the amplification mix for two hours at 37°C in a darkened humidified chamber. After washing with 1x Buffer B for 10 minutes followed by a 1 minute wash with 0.01X buffer B, the cells were stained with DAPI for 2 minutes and mounted onto microscope slides using mounting media. Cells were then viewed under a fluorescence microscope.

### Statistical analysis

Non-paired Student's *t*-test was used to compare the means of two groups and *P* < 0.05 was considered statistically significant.

## SUPPLEMENTARY MATERIALS FIGURES


