# Supplementary material for: Gelsolin-mediated activation of PI3K/Akt pathway is crucial for hepatocyte growth factor-induced cell scattering in gastric carcinoma
Source: Oncotarget. 2016 Apr 5;7(18):25391–407. doi: 10.18632/oncotarget.8603 (PMC5041912; doi:10.18632/oncotarget.8603)
Supplement: Supplementary file 1 [file oncotarget-07-25391-s001.pdf]

Gelsolin-mediated activation of PI3K/Akt pathway is crucial for hepatocyte growth factor-induced cell scattering in gastric carcinoma

Supplementary Material

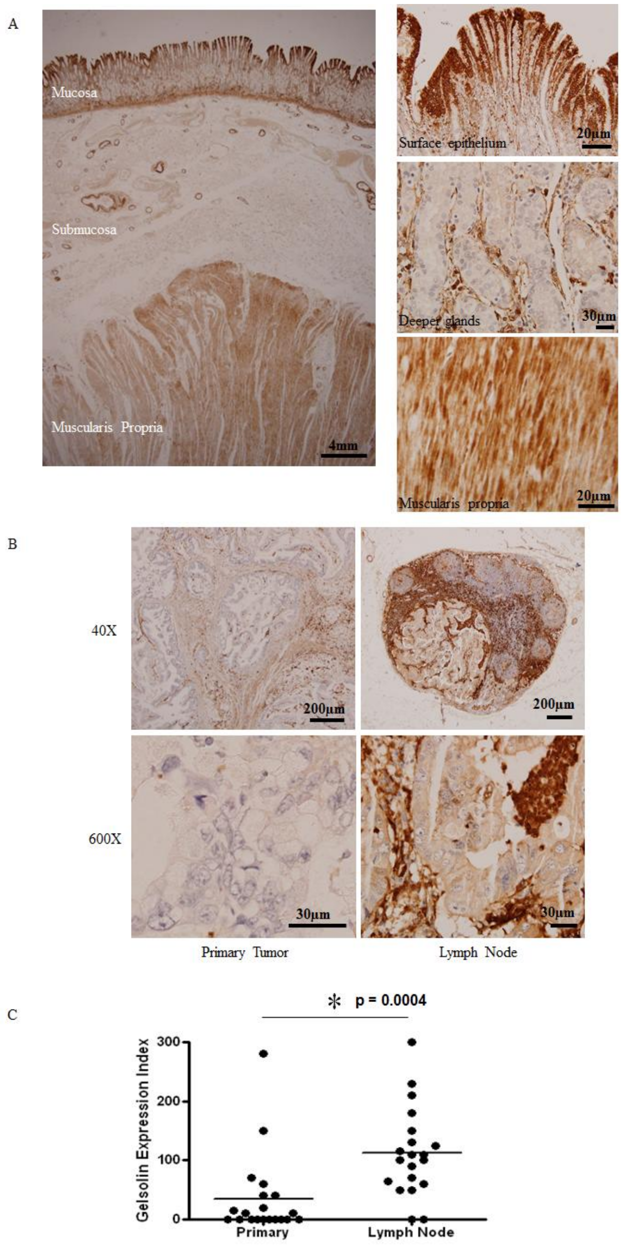

D

Gelsolin Expression	No change	Decrease	Increase
Lymph Node Metastasis/Primary tumor	0 (0%)	2 (10%)	18 (90%)

Supplementary Figure 1.

(A) IHC staining of gelsolin in normal gastric tissue. Left panel: Full thickness of normal gastric tissue. Right panel: Gelsolin staining of surface epithelium of mucosa (upper), gastric glands of mucosa (middle), and muscularis propia (lower).

(B) IHC staining of gelsolin expression in primary tumor versus lymph node metastases from 20 intestinal tumors (x40 and x600 magnification).

(C) Gelsolin expression index in primary tumor and lymph node metastases from 20 intestinal tumors. P=0.0004. Score was expressed as the sum of (intensity x corresponding % positivity), where intensity ranges from 0 (no observable staining) to 3 (intense staining). Paired *t*-test was used to compute p-value shown.

(D) Gelsolin expression between lymph node metastasis and matched primary tumor was compared in 20 intestinal-type tumors. The ratio of gelsolin expression in lymph metastasis/primary tumor was calculated and expressed as increased gelsolin expression if ratio > 1, and decreased gelsolin expression if ratio<1.

(E) IHC staining of gelsolin (left panel) and IgG (right panel) were performed using tissue samples from the same patient.

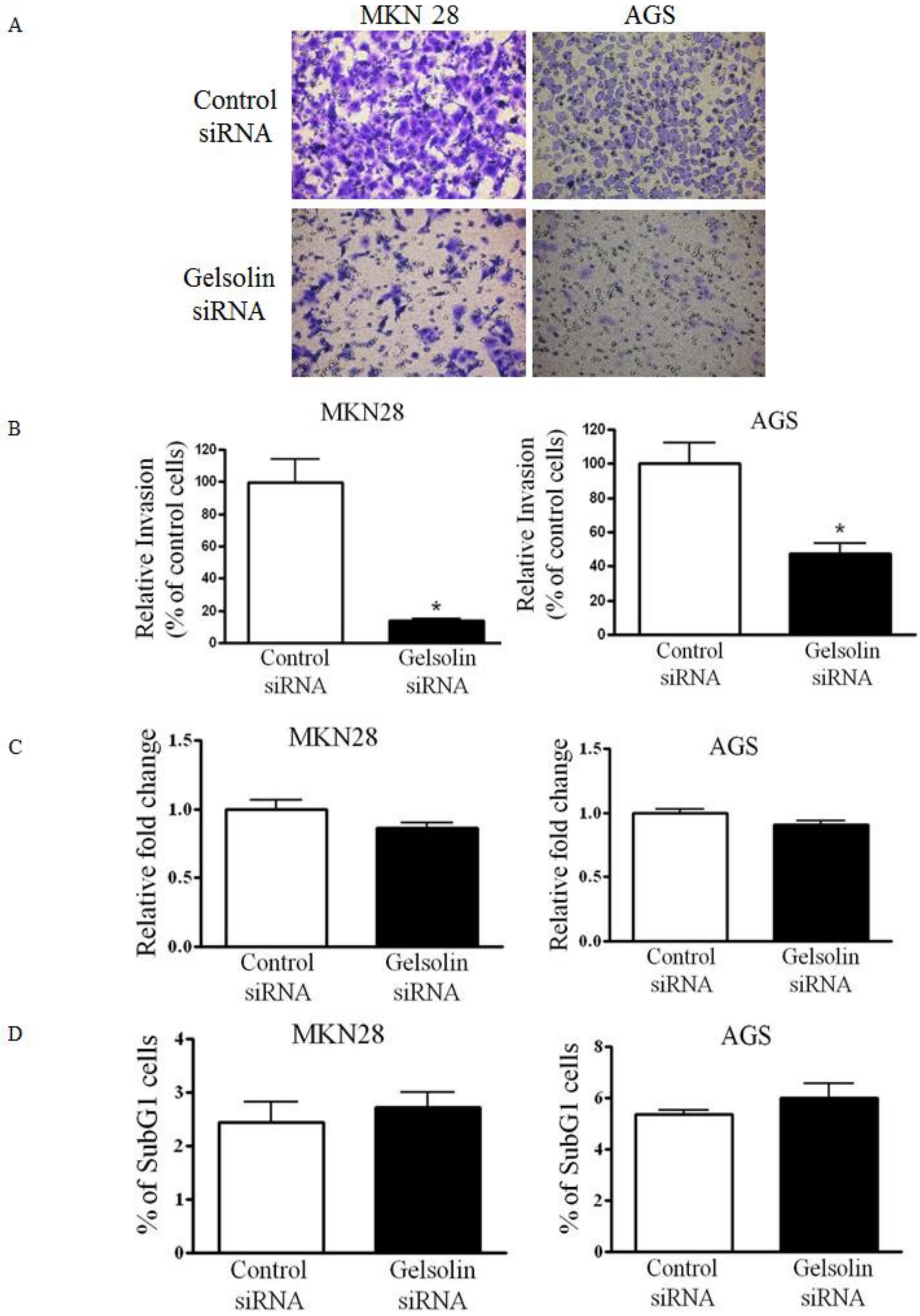

A
MKN 28
AGS
Control siRNA
Gelsolin siRNA
B
MKN28
AGS
Relative Invasion (% of control cells)
120
100
80
60
40
20
0
*
Control siRNA
Gelsolin siRNA
C
MKN28
AGS
Relative fold change
1.5
1.0
0.5
0.0
Control siRNA
Gelsolin siRNA
D
MKN28
AGS
% of SubG1 cells
4
3
2
1
0
8
6
4
2
0
Control siRNA
Gelsolin siRNA

Supplementary Figure 2.

(A) Invasion assay of MKN28 and AGS cells upon transfection of control scrambled RNA or siGelsolin RNA. Images are taken at x100 magnification. Images are representatives from three independent experiments.

(B) Left: Number of MKN28 cells that invaded through invasion chamber were stained and counted. Results are expressed as percentage increases from control cells. Right: Number of MKN28 cells that invaded through invasion chamber were stained and counted. Results are expressed as percentage increases from control cells ± SD, n=3, *$p<0.05$ vs. control.

(C) Left: Cell proliferation BrdU ELISA assay in MKN28 cells upon silencing of gelsolin. Right: Cell proliferation BrdU ELISA assay in AGS cells upon silencing of gelsolin. Values represent mean ± SD, n=3.

(D) Left: Flow cytometric analysis of PI staining in fixed MKN28 cells upon silencing of gelsolin. Right: Flow cytometric analysis of PI staining in fixed AGS cells upon silencing of gelsolin. Values represent mean percentages of cells in sub-G1 fraction ± SD, n=3.

A

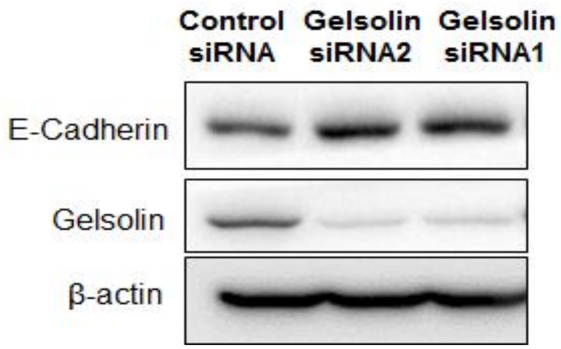

Supplementary Figure 3.

(A) MKN28 cells were transfected with control siRNA or two siRNA sequences targeting gelsolin for 48h. E-Cadherin expression was analyzed by western blot after transfection.

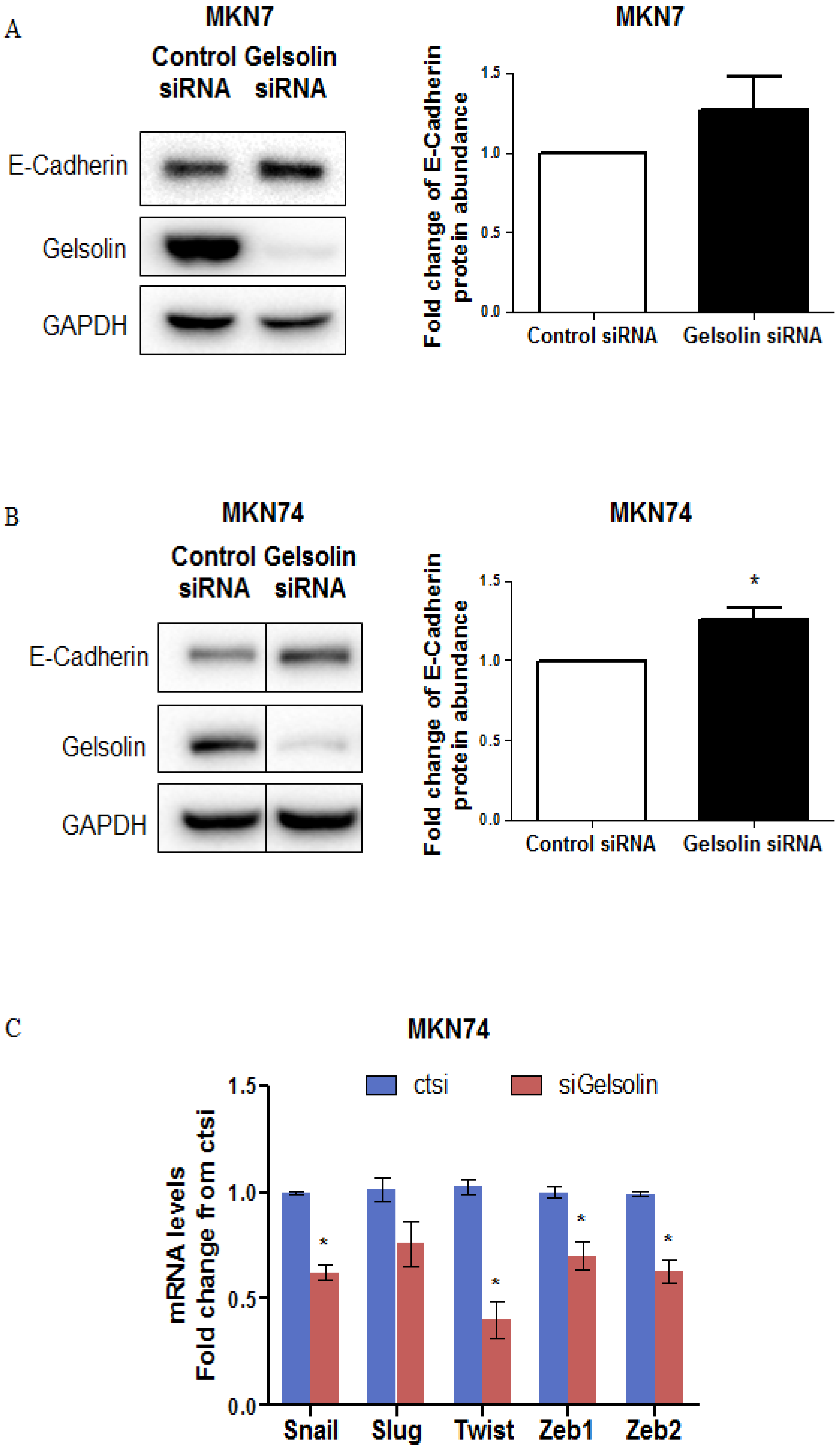
A
MKN7
Control siRNA
Gelsolin siRNA
E-Cadherin
Gelsolin
GAPDH
MKN7
Fold change of E-Cadherin protein abundance
1.5
1.0
0.5
0.0
Control siRNA
Gelsolin siRNA
B
MKN74
Control siRNA
Gelsolin siRNA
E-Cadherin
Gelsolin
GAPDH
MKN74
Fold change of E-Cadherin protein abundance
1.5
1.0
0.5
0.0
*
Control siRNA
Gelsolin siRNA
C
MKN74
ctsi
siGelsolin
mRNA levels
Fold change from ctsi
1.5
1.0
0.5
0.0
Snail
Slug
Twist
Zeb1
Zeb2
*

Supplementary Figure 4

(A) Left: Western blot analysis of MKN7 cells transfected with control siRNA or gelsolin siRNA for 72h. Right: Demsitometric analysis of E-Cadherin protein levels normalized to GAPDH protein levels after transfection in MKN7 cells.

(B) Left: Western blot analysis of MKN74 cells transfected with control siRNA or gelsolin siRNA for 72h. Right: Demsitometric analysis of E-Cadherin protein levels normalized to GAPDH protein levels after transfection in MKN74 cells. Values represent mean ± SD, n=3, *$P<0.05$ vs. control.

(C) Snail, Slug, Twist, Zeb1 and Zeb2 mRNA levels in MKN74 cells after transfection with control siRNA or gelsolin siRNA for 72h, normalized to GAPDH mRNA levels. Values represent mean ± SD, n>3, *$P<0.05$ vs. control siRNA transfected cells.

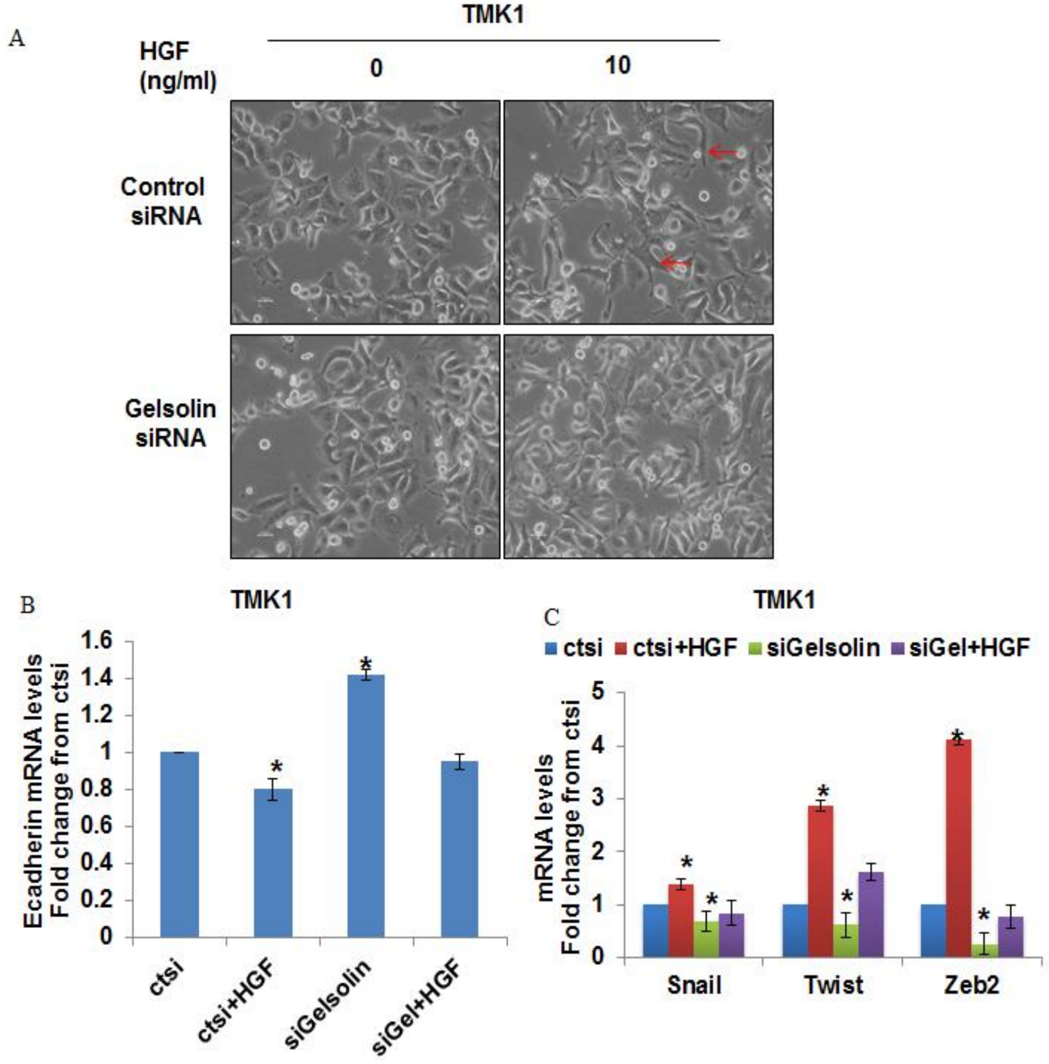

Supplementary Figure 5.

(A) Light microscopy images of TMK1 cells transfected with ctsi or siGelsolin and treated with HGF for 48h (x100 magnification). Images are representatives from three independent experiments.

(B) E-cadherin mRNA levels in TMK1 cells transfected with ctsi or siGelsolin and treated with HGF for 48h, normalized against GAPDH mRNA levels. Values represent mean ± SD, n=3, **P*<0.05 vs. control.

(C) Snail, Twist and Zeb2 mRNA levels in TMK1 cells transfected with ctsi or siGelsolin and treated with HGF for 48h, normalized against GAPDH mRNA levels. Values represent mean ± SD, n=3, **P*<0.05 vs. control.

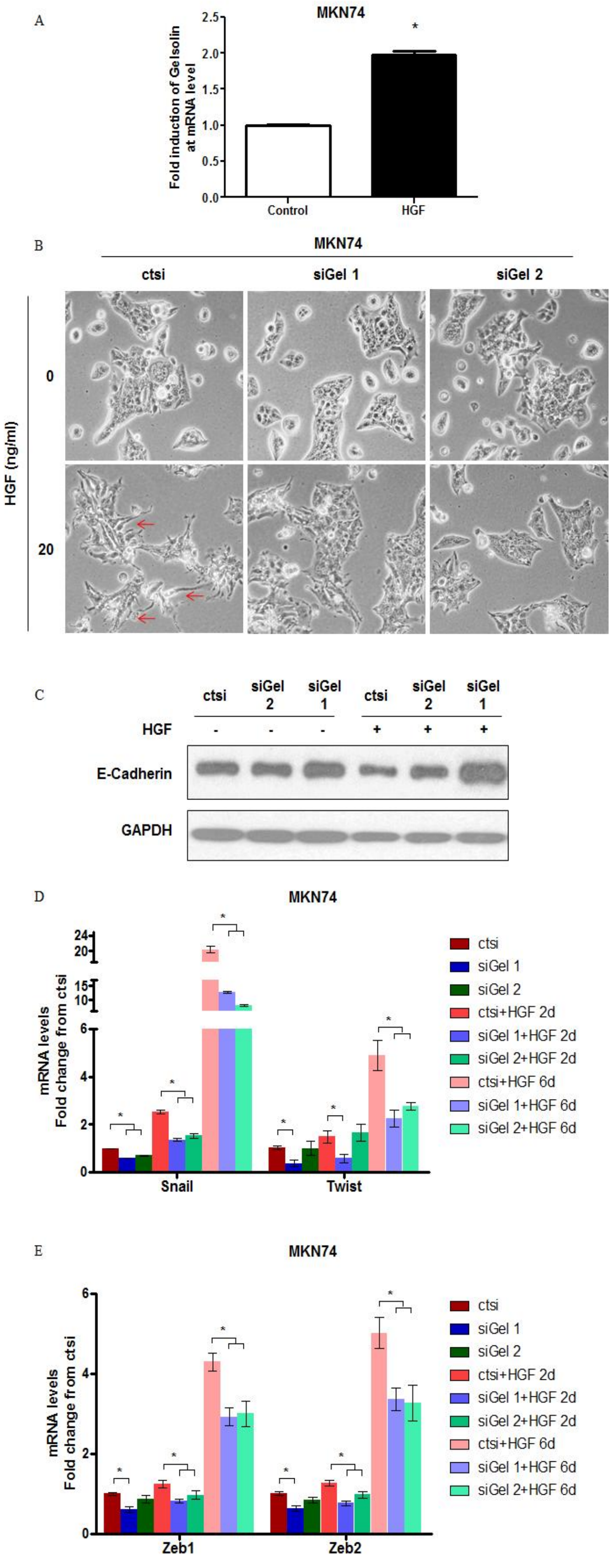
A
MKN74
Fold induction of Gelsolin at mRNA level
Control
HGF
B
MKN74
ctsi
siGel 1
siGel 2
HGF (ng/ml)
0
20
C
ctsi
siGel 2
siGel 1
HGF
E-Cadherin
GAPDH
D
MKN74
mRNA levels Fold change from ctsi
Snail
Twist
ctsi
siGel 1
siGel 2
ctsi+HGF 2d
siGel 1+HGF 2d
siGel 2+HGF 2d
ctsi+HGF 6d
siGel 1+HGF 6d
siGel 2+HGF 6d
E
MKN74
mRNA levels Fold change from ctsi
Zeb1
Zeb2

Supplementary Figure 6.

(A) Gelsolin mRNA levels in MKN74 cells treated with 20ng/ml HGF for 48h. Gelsolin levels were normalized against GAPDH levels. Values represent mean ± SD, n=3, *$P<0.05$ vs. control.

(B) Light microscopy images of MKN74 cells transfected with ctsi or two gelsolin siRNAs (siGel 1 and siGel 2) and treated with 20ng/ml HGF for 5 days (x200 magnification). Images are representatives from three independent experiments.

(C) Western blot of MKN74 cells transfected with ctsi or two gelsolin siRNAs (siGel 1 and siGel 2) and treated with HGF for 24h and blotted for E-Cadherin protein expression.

(D) Snail, Twist, (E) Zeb1 and Zeb2 mRNA levels in MKN74 cells transfected with ctsi, siGel 1 or siGel 2 and treated with HGF for 2 days (48 hours) or 6 days. Level of respective gene was normalized against GAPDH mRNA levels. Fold changes of mRNA compared to ctsi transfected cells without HGF treatment were presented. Value represent mean± SD, n=4, *$P<0.05$ vs. control.

A

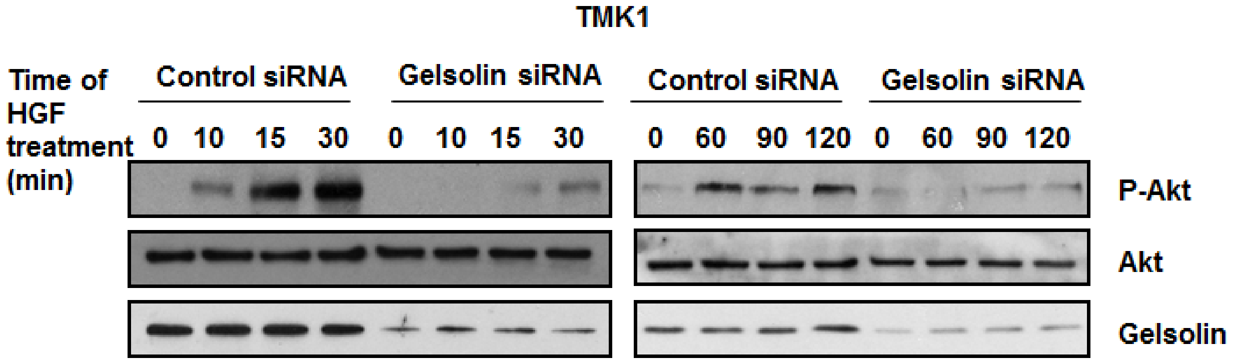

Supplementary Figure 7.

(A) Western blot of TMK1 cells transfected with ctsi or siGelsolin before treatment with HGF for 0-120 min and analyzed for gelsolin, phosphorylated Akt and Akt protein levels.
